# EEG Microstate Correlates of Emotional Processing in Patients with Alcohol use Disorder and Healthy Controls: An Exploratory Resting-EEG Study

**DOI:** 10.1007/s10548-026-01180-z

**Published:** 2026-03-27

**Authors:** Oliver B. Affentranger, Thomas Koenig, Franz Moggi, Leila M. Soravia, Ingo Butzke, Hallie M. Batschelet, Raphaela M. Tschuemperlin, Maria Stein

**Affiliations:** 1 University Hospital of Psychiatry and Psychotherapy, Translational Research Center, University of Bern, Bern, Switzerland; 2 Clinic Suedhang, Center for Treatment of Addictive Disorders, Südhang 1, Kirchlindach, 3038 Switzerland; 3 Psychiatriezentrum Münsingen, Hunzigenstrasse 1, Münsingen, 3110 Switzerland; 4 Department of Clinical Psychology and Psychotherapy, Institute of Psychology, University of Bern, Fabrikstrasse 8, Bern, 3012 Switzerland; 5 Psychiatriezentrum Wetzikon, Bahnhofstrasse 196, Wetzikon, 8620 Switzerland

**Keywords:** Alcohol use disorders, Microstate, Emotional processing, EEG, Resting state

## Abstract

Emotion processing is a key domain in alcohol use disorder (AUD). Given the neuronal basis of emotion regulation and the neurophysiological deviations observed in AUD patients the present exploratory study investigates EEG Microstate (MS) correlates of emotional processing in patients with AUD and healthy controls (HC). Emotions, moods and emotion regulation competencies were assessed in 29 patients with AUD and 22 HC using self-report. Additionally, a 64-channel resting-state electroencephalography (EEG) was recorded for each individual and a microstate analysis yielding 7 classes (A-G) was performed. The effects of the factors *group* (patients with AUD vs. HC), *emotion/emotion regulation* (from self-report scales) and MS *class* (A-G) on the MS feature *contribution* were analyzed using linear models. Three-way interactions were observed for “awareness of feelings” (Bonferroni-corrected; *F*(6, 329) = 3.75, *p* = .001) and “shame” (*F*(6, 322) = 2.42, *p* = .026). Level of awareness of feelings correlated positively with MS C in HC, while an inverse correlation was found for MS D. No such association between awareness of feelings and MS C and D was observed in patients with AUD who rather displayed significantly higher overall MS C values than HC (*t*_*Ratio*_(343) = -3.13, *p* = .002). The present study provides initial evidence that neuronal activity, as reflected in MSs, differs in relation to emotion processing between patients with AUD and healthy individuals. The results indicate that MS C and D might be neurophysiological markers for awareness of feelings in HC, while such an association is blunted in patients with AUD.

**Supplementary Information:**

The online version contains supplementary material available at https://doi.org/10.1007/s10548-026-01180-z.

## Introduction

Given the prevalence of 5.1% (World Health Organization 2019) and detrimental effects on individual and societal levels, alcohol use disorder (AUD) is a severe public health concern (Rehm 2011; Collins et al. 2011). Effects of specialized treatments are limited by a considerable proportion of patients who relapse after treatment. In current conceptualizations of AUD, maladaptive negative emotionality is one of the key domains (Kwako et al. 2016, 2017; Drossel et al. 2023; Boness et al. 2021; Yucel et al. 2019), potentially elevating the relapse risk (e.g. Falk et al. 2008; Gamble et al. 2010; Sinha et al. 2009; Witkiewitz and Villarroel 2009). Consequently, research has sought to elucidate the role of emotional processing and emotion regulation in the development, maintenance, and treatment of AUD (Colder and Chassin 1993; Khantzian 1997; Berking et al. 2011). Emotion regulation involves processes, which serve to monitor and appraise emotions as well as to regulate the intensity and duration of emotional reactions (Thompson 1994; Gross 2014; Berking 2007). It can be defined as a multidimensional construct (Gratz and Roemer 2004; Berking and Znoj 2008), comprising processes ranging from the awareness of emotions over the ability to modify negative emotions to compassionate self-support in distressing situations (Berking et al. 2012). Emotion regulation is key to mental health (Gross and Muñoz 1995; Berking et al. 2010) and of transdiagnostic relevance (Cal et al. 2010; Fernandez et al. 2016) as it has been linked to numerous psychopathological symptoms and psychiatric disorders (Berking et al. 2012; Berking and Wupperman 2012). Lower ability for emotion regulation has also been observed in patients with AUD (Berking et al. 2011; Filipek et al. 2010), where individual levels of emotion regulation, especially the ability to tolerate negative emotions, predicted drinking during as well as after treatment (Berking et al. 2011; Filipek et al. 2010). Studies on the related construct of emotional intelligence (Salovey and Mayer 1990) demonstrated a negative association between emotional intelligence and alcohol-related problems (Riley and Schutte 2003) and showed that emotional intelligence moderates the association between negative emotions and craving (Uva et al. 2010). Emotion regulation is neuronally based (e.g. Etkin et al. 2015) and patients with AUD show various neurophysiological modifications compared to HC (Zhang et al. 2021). Studying the neuronal activity underlying emotion regulation in patients with AUD might thus reveal deviations in the neuronal anchorage of this relevant – and relapse-predicting - process in AUD.

Resting-state electroencephalography (EEG) provides a reliable way to measure electrical activity of brain networks with high temporal resolution and is characterized by stability across time (Napflin et al. 2007) and intraindividual specificity (Zietsch et al. 2007; Schmid et al. 2000). EEG studies on AUD have characterized this disorder through elevated theta- and beta-power (Campanella et al. 2009; Bauer 1994; Costa and Bauer 1997; Herrera-Diaz et al. 2016), although the effects in the beta band might be less pronounced in female patients (Rangaswamy et al. 2002). Furthermore, prolonged abstinence and an increased relapse risk have been linked to changes in slow-wave activity, alpha, and beta power (Saletu-Zyhlarz et al. 2004; Winterer et al. 1998). In HC, EEG research on emotional processing has linked subprocesses such as attention control in emotional contexts (Tortella-Feliu et al. 2014; Angelidis et al. 2016; Putman et al. 2010, 2014), emotion differentiation (Wang et al. 2020), and distraction as an emotion regulation strategy (Kobayashi et al. 2020) to the ratio between slow and fast EEG wave amplitudes. More specifically, studies in healthy individuals indicate that a higher theta/beta ratio in resting EEG is associated with greater difficulties in emotion regulation (Wang et al. 2020; Tortella-Feliu et al. 2014). When linking these findings to the aforementioned EEG studies in patients with AUD, they suggest that the neurophysiological reflection of impaired emotional processing and AUD seem to overlap at least in part. However, to the best of our knowledge, EEG research into emotional processing in AUD is yet missing and could help to elucidate the neurophysiological basis of this important process.

A global approach to the analysis of resting-state EEG is microstate (MS)-analysis, which describes the continuous EEG through a series of meta-stable topographies, each of them representing a distinct generator network being active in the brain (Michel et al. 2009; Michel and Koenig 2018; Koenig et al. 2002). A first bulk of literature has demonstrated the occurrence of four canonical topographies (MS A, B, C, D; Koenig et al. 2002), which could explain up to 80% of global variance in a series of independent studies (e.g. Tomescu et al. 2014; Michel and Koenig 2018). More recently, research has extended these findings to the description of up to seven MS (MS A-D and E, F, G) best describing resting state EEG data (Custo et al. 2017). By analyzing the association of specific conditions and/or individual characteristics with MS characteristics such as duration (how long is a given MS), occurrence (how often does a MS occur), contribution (how much of the data is best described by this MS) and mean global field power (overall amplitude of this MS), the different MS have been related to specific neuronal networks (e.g. Khanna et al. 2015; Schiller et al. 2019). According to a systematic review of 50 studies (Tarailis et al. 2024), MS A is associated with auditory and visual processing and subject’s arousal, and MS B with visual processing in relation to self, self-visualization and autobiographical memory. MS C is linked to neuronal networks for processing personally significant information, self-reflection and self-referential internal mentation, MS D to executive functioning, and MS E to the processing of interoceptive and emotional information and to the salience network. MS F is considered to reflect Default Mode Network activity and processing personally significant information, mental simulations and theory of mind, while MS G is potentially related to the somatosensory network.

With respect to substance use disorders, Chen et al. (2020) reported reduced duration of MS A and B in patients with methamphetamine use disorder (MUD), with the size of this effect being positively related to the frequency of methamphetamine use. Furthermore, Schiller et al. (2021) demonstrated that acute alcohol intoxication was related to reduced contribution of MS C, while at the same time contribution of MS B increased.

While MS-research focusing on emotional processing is rare, one might assume that MS C, which is linked to self-reflection and self-referential internal mentation, might be related to emotional processing. Consistent with this assumption, MS C was negatively related to the affective lability score (Affective Lability Scale, Harvey et al. 1989) and to the presence of borderline personality disorder (Deiber et al. 2024). Furthermore, MS D and its connection to executive functions may be linked to emotional processing via coping strategies and other cognitive emotion regulation strategies. Such a link is tentatively supported by findings relating less MS D to positive (vs. negative) emotional valence during stimulation with emotional video material (Shen et al. 2020; Hu et al. 2023). Also, the self-domain of the “Amsterdam Resting-State Questionnaire” (ARSQ; Diaz et al. 2013), which is evaluated with statements such as “I thought about my feelings”, correlated negatively with the duration of MS D (Tarailis et al. 2021). According to the review by Tarailis et al. (2024), MS E was related to processing interoceptive and emotional information and to the salience network. Therefore, MS E should also be linked to emotional processing. However, given that merely six studies have reported significant results for MS E (before relabeling), conclusions should be treated with caution. Finally, as an interesting and more general effect, Kleinert et al. (2022) found that - across the MS classes A, B, C and D - longer MS duration was associated with better self-regulation and inhibition skills. As MS research directly investigating emotional processing in AUD is still lacking, findings on MS C – linked to self-reflection (Tarailis et al. 2024), affective lability (Deiber et al. 2024) and alcohol intoxication (Schiller et al. 2021) – underscore the neurophysiological connection between impaired emotional processing and AUD.

In summary, the neuroscientifically based construct of emotion regulation (Fernandez et al. 2016) is of high relevance, and potentially altered, in AUD (Boness et al. 2021; Berking et al. 2011; Filipek et al. 2010). In combination with measures of emotion regulation, MS analysis of resting-state EEG may advance the description of the neurophysiological anchorage of emotion regulation in AUD. A deeper knowledge of the neurophysiological anchoring of emotion regulation in patients with AUD furthers our scientific understanding of this key process. The present study integrates resting-state EEG measurements with self-report data on emotional processing to investigate the neurophysiological dynamics of emotional processing in patients with AUD and HC.

## Methods

### Participants

A total of *N* = 53 German speaking subjects (31 patients with AUD, 22 HC) participated in the study. Two patients with AUD had to be excluded due to EEG-specific exclusion criteria (see *2.4.1 EEG recording and data reduction*), leaving a final sample of 29 patients with AUD and 22 HC (*N* = 51; 14 females, 37 males, mean age 43.7 (SD = 10.9, range: 25 to 60). Patients with AUD (7 females, 22 males, mean age 43.8 (SD = 10.8, range: 25 to 60) were recruited at the beginning of their inpatient treatment in two specialized treatment centers for severe AUD in Switzerland. All patients had completed detoxification prior to treatment admission. Inclusion criteria for patients were the primary diagnosis of AUD according to the fifth edition of the Diagnostic and Statistical Manual of Mental Disorders’ (American Psychiatric Association 2013), at least three weeks of alcohol abstinence (prior to EEG measurement) and being between 18 and 60 years old. The exclusion criteria were the use of sedatives or opioids (e.g., benzodiazepines, methadone), the presence of another psychiatric axis-I disorder other than AUD as a primary diagnosis (comorbidities allowed, as long as AUD was the main diagnosis), another severe substance use disorder other than nicotine (≥ 25 points per substance on the “Drug Use Disorders Identification Test” (DUDIT); Berman et al. 2005), diagnosed neurocognitive problems (e.g., Korsakoff syndrome), an acute somatic illness (e.g., flu), and insufficient German language skills.

HC (7 females, 15 males, mean age 43.6 (SD = 11.6, range: 25 to 58) were recruited through local advertisement and the study team’s personal contacts. Inclusion criteria were being aged between 18 and 60 years, no diagnosis of AUD in medical history and non-hazardous drinking (< 8 points on the “Alcohol Use Disorders Identification Test” (AUDIT); Babor et al. 2001), and no signs for clinically relevant psychopathological symptoms (GSIt-value ≤ 63 in the “Brief Symptom Check List” (BSCL); Derogatis 1993). Exclusion criteria were an ongoing treatment for a psychiatric disorder, the intake of psychotropic drugs, treatment for substance use disorder, problematic substance use (except nicotine; ≥ 8 points per substance in “DUDIT”), neurocognitive problems, poor health conditions, insufficient German language skills, hearing impairment, and the presence of AUD in first-degree relatives.

### Procedure and Questionnaires

The present study is a substudy of the INTRA-study, for which full details are described elsewhere (Tschuemperlin et al. 2019). Within the first two weeks of inpatient treatment, patients with AUD completed sociodemographic questions and baseline measurement (see Stein et al. 2023 for details), including the alcohol use disorders identification test (AUDIT, Babor et al. 2001) and the self-report instrument for the assessment of emotion-specific regulation skills (ERSQ-ES; Ebert et al. 2014) and self-report measure for the assessment of emotion regulation skills (SEK-27; Berking and Znoj 2008) to assess current feelings, moods and emotional competencies. Three to four weeks after treatment onset, a resting-state EEG was recorded, and patients completed a set of questionnaires before other components of the INTRA-study were performed (Tschuemperlin et al. 2019). HC completed a telephone screening prior to the EEG measurement to assess eligibility. If eligible, HC were invited to the EEG lab, where they filled out sociodemographic and baseline questionnaires, including ERSQ-ES, SEK-27 and AUDIT, and completed the EEG measurement the same day.

All subjects gave written informed consent prior to participation and received monetary compensation for participation. Procedures were approved by the local Swiss ethics committee (No.: 2016_000988) and registered on ClinicalTrials.gov (NCT02968537).

#### Assessment of Emotion and Emotion Regulation

Emotions and emotion regulation were assessed using part A of the self-report instrument for the assessment of emotion-specific regulation skills (ERSQ-ES; Ebert et al. 2014) and the self-report measure for the assessment of emotion regulation skills (SEK-27; Berking and Znoj 2008) questionnaires. All items were answered on a 5-point rating scale ranging from 0 (not at all) to 4 (first part: very much; second part: almost always).

The ERSQ-ES (Ebert et al. 2014), of which only Part A was used, seeks to assess emotions and moods of the individuals. It consists of 50 items (including the 20 items from the PANAS (Watson et al. 1988), which are grouped to form two total scales (positive affect and negative affect) and nine subscales (stress, anxiety, anger, sadness, depressiveness, shame, coping emotions, positive affect PANAS, negative affect PANAS). For the entire ERSQ-ES (incl. Part B), the internal consistencies range between Cronbach’s α = 0.67 and 0.97 and the retest reliabilities (r_tr_; 4-week interval) between r_tr_ = 0.41 and r_tr_ = 0.70. The construct validity is supported by expected factor loadings, hypothesis-confirming differences between the control and clinical samples and expected correlations with related constructs (Ebert et al. 2014).

The SEK-27 (Berking and Znoj 2008) measures the emotion processing and regulation. It consists of 27 items which are grouped to form one total scale (emotion regulation) and nine subscales (awareness of feelings, body sensation of feelings, clarity of feelings, understanding of feelings, acceptance of feelings, resilience, readiness to confront, self-support, regulatory competence). The internal consistencies range between Cronbach’s α = 0.72 and 0.81 (except for the scale “acceptance”, Cronbach’s α = 0.68). The retest reliabilities lie between r_tr_ = 0.48 and 0.74. The substantial correlation with measures associated with emotional competence and the factorial validity, which largely confirms the assumed basic structure, support the validity (Berking and Znoj 2008).

### Statistical Analyses of Questionnaires

Using jamovi (The jamovi project 2023) demographic differences between patients with AUD and HC were tested with independent t-tests (and χ^2^ for gender distribution), and the ERSQ-ES and SEK-27 scores of patients and HC were investigated with Welch’s T-tests as recommended by Rasch et al. (2011) and Ruxton (2006).

### Electrophysiological Data

#### EEG Recording and Data Reduction

Resting-state EEG data was recorded with the EEG system “actiCap Plus” (2019, Version 2.0, Gilching, Germany: Brain Products GmbH), BrainAmps, and the “Brain Vision Recorder” (2019, Version 2.0, Gilching, Germany: Brain Products GmbH) from 64 active electrodes placed according to the extended 10/10 system. The data was digitalized with a sampling rate of 500 Hz, all impedances were kept below 20 kΩ, online filters were set to 0.016 Hz (high pass) and 250 Hz (low pass), and FCz was used as the online reference electrode. Resting-state EEG was recorded for at least 5 min with eyes closed (interrupted in the middle by at least a 30-second-epoch of eyes open). For the EEG data preprocessing the software “Brain Vision Analyzer” (2019, Version 2.2, Gliching, Germany: Brain Products GmbH) was used. Biological artifacts (eye movements, muscle artifacts) were removed by using “Independent Component Analysis” (ICA): defective or very noisy electrode data was interpolated, and remaining artifacts (e.g., strong movements, sweating) were removed manually. All data was then recomputed against average reference and the data from the eyes-closed recording was segmented in 2-second-epochs (data from eyes-open recording was excluded from further analyses). To reduce EEG-variability, only subjects from whom at least 20 artifact-free epochs (i.e. 40 s) of clean data could be obtained were included in the analyses (Gasser et al. 1985). One participant had to be excluded because this criterion was not met. Furthermore, another subject identified by the open-source Software „Randomization Graphical User interface“ (Ragu; Koenig et al. 2011) as an EEG outlier was excluded from further analyses. Accordingly, two of the original 31 patients with AUD were excluded from the analysis, leaving *N* = 29 patients in the final analytic sample (see also *2.1 Participants*). In the final sample of 51 participants, a mean of 77 (range: 26–103) artifact-free, eyes-closed, 2-second-epochs per subject was extracted for further analyses. Finally, the EEG data was exported and filtered (2–20 Hz) by using the Matlab toolbox “EEGLAB” (Delorme and Makeig 2004).

#### Microstate (MS) Analysis

##### Cluster Analyses

Cluster analyses were run using the “MICROSTATELAB-plugin” (Nagabhushan Kalburgi et al. 2024) for the Matlab toolbox “EEGLAB” and followed well-established procedures. First, individual MS cluster maps were obtained using a modified k-means algorithm (Pascual-Marqui et al. 1995), applied to all maps at local GFP maxima of each individual EEG, using all available artifact-free segments per subject. Next, group-specific mean maps (AUD & HC) were calculated from the individual MS cluster maps, and the obtained group mean MS maps were combined into grand mean MS template sets. Finally, the obtained sets of grand mean MS template maps were labeled according to their similarity to the template maps published by Custo et al. (2017), and the individual MS cluster maps were ordered and labeled according to their similarity to the grand mean template maps. The entire procedure of obtaining these grand-mean MS template maps was conducted with a k ranging from 4 to 7 to allow for an informed decision about the final choice of the number of clusters at a later analyses stage. Details and justifications for the entire procedure can be found in Nagabhushan Kalburgi et al. (2024).

The rationale for our choice of the number of MS classes was motivated by the goal of minimizing the confounding effects of topographic differences upon the analysis of MS parameters (Murphy et al. 2024). We thus compared the two groups’ individual MS cluster maps using TANOVAs for all (k = 4 to 7) cluster solutions and gave preference to the first (smallest k) solution that showed no significant group differences in these TANOVAs. The grand mean template maps of the thus selected solution were then used to extract the standard MS temporal features, using assignments to GFP-peak maps only and nearest neighbor interpolation between GFP-peaks. Given the exploratory nature of the study and the entailing problem of multiple testing, we limited the subsequent statistical modeling of the extracted features to MS contribution as this feature seemed best suited to catch the overall picture. For completeness, additional results regarding the MS parameters duration, occurrence and meanGFP are given in the Online Resource.

##### Statistical Analysis of Microstate (MS) Quantifiers

First, a mixed model with the factors *class* and *group* was calculated to analyze the scale-independent effects and to investigate pairwise comparisons (group contrasts) of the estimated marginal means. Next, to explore the variation of MS contribution (dependent variable) in dependence of the scores on the specific ERSQ-ES/SEK-27 scales, the specific MS classes (A-G) and the groups (AUD vs. HC), a three-factorial linear mixed model (LMM; fitting: restricted maximum likelihood) was calculated for each of the 9 subscales of the ERSQ-ES and for each of the 9 subscales of the SEK-27 (fixed within effect: *class*, fixed between effects; *group* & *ERSQ-ES/SEK-27 scale*, random effect: *Subject*). For the follow-up investigation of significant three-way interactions, representing the main focus of the analyses, two further LMMs (fixed effects: *Class* & *ERSQ-ES/SEK-27 scale* / random effect: *Subject*) were calculated specifically for both the AUD- and the control group. In case of significant two-way interactions (*class* x *ERSQ-ES/SEK-27 scale*) within a specific data set (AUD or HC), further follow-up analyses were conducted in the form of Pearson correlations. Here, for each MS class, the contribution value was correlated with the score of the specific ERSQ-ES/SEK-27 scale in the data set of interest (patients with AUD or HC). In all MS analyses, a two-sided significance level of α = 0.05 was used and a Bonferroni-correction was made. All significant Bonferroni-corrected p-values are reported. For completeness, the significant Bonferroni-uncorrected p-values are also reported but should be interpreted with caution. The p-values for the scale-independent effects as well as for the fixed effects were determined using a Type III Analysis of Variance (ANOVA) table with Satterthwaite’s degrees of freedom method.

## Results

### Sample Description

We included 29 patients with AUD and 22 HC in the analyses. As can be seen in Table 1, the groups did not differ in terms of sex, age, years of education, marital status, and emotion-related variables but did significantly differ in living situation, employment, and AUDIT scores. All subjects’ existing medication was checked, yielding *N* = 10 patients taking antidepressants and *N* = 3 patients taking antipsychotics in very low dosages for sleep-inducing effects. The patients had suffered from AUD for an average of 10.66 years, with a range from 2 to 29 years. Furthermore, the patients fulfilled an average of 7.64 of the eleven DSM-5 criteria, with no individual fulfilling fewer than 4 criteria. With regard to group-specific mean differences in the ERSQ-ES/SEK-27 scales (see Table 2), the AUD group and the control group differed significantly on the scales “depressiveness” and “understanding of feelings”, with patients with AUD showing higher depressiveness and lower understanding of feelings.

**Table 1:** Comparison of demographic, alcohol-related and emotion-related variables in patients with AUD and healthy controls

	N	Patients withAUD	HC	t orχ2	p
**Demographics**					
*Sex*	51				
Male / female		22 / 7	15 / 7	0.37	0.543
*Age*	49				
Mean (SD)		43.75 (10.77)	43.57 (11.59)	0.06	0.956
Range		25–60	25–58		
*Years of education*	49				
Mean (SD)		13.82 (2.57)	15.43 (3.14)	-1.91	0.063
Range		9–20	12–23		
*Living situation*	49			16.5	0.002**
Alone		46.4%	4.8%		
With partner		42.9%	81.0%		
With parents		7.1%	0.0%		
With children		3.6%	0.0%		
With roommates		0.0%	14.3%		
*Marital status*	49			6.75	0.080
Single		53.6%	28.6%		
Married, living together		28.6%	52.4%		
Married, living separately		0.0%	0.0%		
Concubinate / in a serious relationship		0.0%	9.5%		
Divorced		17.9%	9.5%		
*Employment*	49			13.3	0.001**
Yes, full-time		39.3%	71.4%		
Yes, part-time		14.3%	28.6%		
No		46.4%	0%		
**Alcohol-related variables**					
*AUDIT total score*	49				
Mean (SD)		26.16 (5.22)	4.52 (2.14)	19.84	< 0.001***
Range		17–36	0–7		
*Years of AUD*	28				
Mean (SD)		10.66 (7.39)	-	-	-
Range		2–29	-	-	-
*DSM-5 criteria of eleven fulfilled*	28				
Mean (SD)		7.64 (2.00)	-	-	-
Range		4–11	-	-	-

Number of participants (N) varies between different variables due to unanswered items.

AUD: Alcohol Use Disorder; AUDIT: Alcohol Use Disorder Identification Test; DSM: Diagnostic and Statistical Manual of Mental Disorders; HC: Healthy controls; SD: Standard deviation.

*Note on probability.* * *p* < .05. ** *p* < .01. *** *p* < .001.

**Table 2:** *Comparison of ERSQ-ES/SEK-27 scores in patients with AUD and healthy controls.*

	N	Patients with AUD	HC	Welch’s t	p
**Feelings and moods**					
*Stress*	50			0.36	0.72
Mean (SD)		1.36 (1.08)	1.26 (0.89)		
Range		0.00–4.00	0.00–3.00		
*Anxiety*	49			0.68	0.50
Mean (SD)		1.05 (0.88)	0.89 (0.72)		
Range		0.00-3.33	0.00-2.67		
*Anger*	51			-0.91	0.37
Mean (SD)		0.74 (0.74)	0.91 (0.62)		
Range		0.00-2.33	0.00–2.00		
*Sadness*	49			1.59	0.12
Mean (SD)		1.26 (0.87)	0.92 (0.60)		
Range		0.00-3.67	0.00-2.33		
*Depressiveness*	49			2.55	0.01*
Mean (SD)		0.89 (0.94)	0.37 (0.48)		
Range		0.00–3.00	0.00-1.33		
*Shame*	49			1.70	0.10
Mean (SD)		0.53 (0.74)	0.26 (0.36)		
Range		0.00-2.67	0.00–1.00		
*Coping emotions*	49			-1.09	0.28
Mean (SD)		2.46 (0.88)	2.68 (0.53)		
Range		0.55–3.82	1.64–3.82		
*Positive affect (PANAS)*	51			-1.31	0.20
Mean (SD)		2.37 (0.98)	2.64 (0.50)		
Range		0.20–3.80	2.00-3.70		
*Negative affect (PANAS)*	51			0.55	0.58
Mean (SD)		0.92 (0.75)	0.82 (0.57)		
Range		0.00-2.60	0.20-2.00		
**Emotional competencies**					
*Awareness of feelings*	51			0.36	0.72
Mean (SD)		2.55 (0.97)	2.47 (0.66)		
Range		0.33-4.00	1.33–3.67		
*Body sensation of feelings*	51			-0.46	0.65
Mean (SD)		2.69 (0.76)	2.79 (0.75)		
Range		1.00-3.67	0.67-4.00		
*Clarity of feelings*	51			-1.79	0.08
Mean (SD)		2.78 (0.85)	3.17 (0.69)		
Range		1.00–4.00	2.00–4.00		
*Understanding of feelings*	51			-2.21	0.03*
Mean (SD)		2.54 (0.94)	3.02 (0.60)		
Range		0.67-4.00	2.00–4.00		
*Acceptance of feelings*	51			-1.31	0.20
Mean (SD)		2.69 (0.90)	2.95 (0.54)		
Range		0.67-4.00	2.00–4.00		
*Resilience*	51			-1.92	0.06
Mean (SD)		2.54 (1.16)	3.03 (0.64)		
Range		0.00–4.00	2.00–4.00		
*Readiness to confront*	51			-0.87	0.39
Mean (SD)		2.74 (0.90)	2.95 (0.89)		
Range		1.00–4.00	1.00–4.00		
*Self-support*	50			0.82	0.39
Mean (SD)		2.70 (0.87)	2.62 (0.54)		
Range		1.00–4.00	1.67-4.00		
*Regulatory competence*	51			-1.00	0.32
Mean (SD)		2.21 (1.02)	2.44 (0.64)		
Range		0.00–4.00	1.33-4.00		

Number of participants (N) varies between different scales due to unanswered items or outlying values.

AUD: Alcohol Use Disorder; HC: Healthy controls; SD: Standard deviation.

*Note on probability.* * *p* < .05. ** *p* < .01. *** *p* < .001.

### Microstate (MS) Results

#### Model Selection

**Fig. 1:**
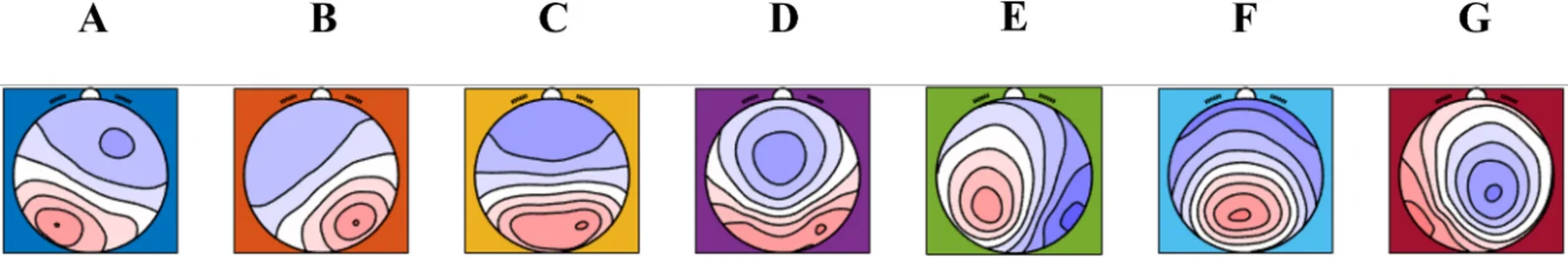
The seven MSs **(A-G)** across all participants (GrandMean maps, perspective from above, nose depicted on the top). Note that in the meta-analysis by Tarailis et al. (2024), the MS classes E and F are interchanged compared to our assignment, which is based on the assignment in the paper by Custo et al. (2017)

The TANOVA results showed that the specific MS maps differed significantly between the groups for the 4-, 5- and 6-class solutions. This indicates that in these solutions, the single MS classes do not capture the same neuronal activating patterns in both groups. Using one of these solutions would thus introduce a systematic bias in the analyses. Only in the 7-class solution, no significant topographic differences were found between the MS of the two groups (*p* > .190, *e.V.* = 13.2%), indicating that the MSs in this solution capture comparable activation patterns in the both groups. Therefore all calculations were based on the grand mean MS maps obtained by the 7-class solution, which is shown in Fig. 1.

#### Scale-independent Effects

**Fig. 2:**
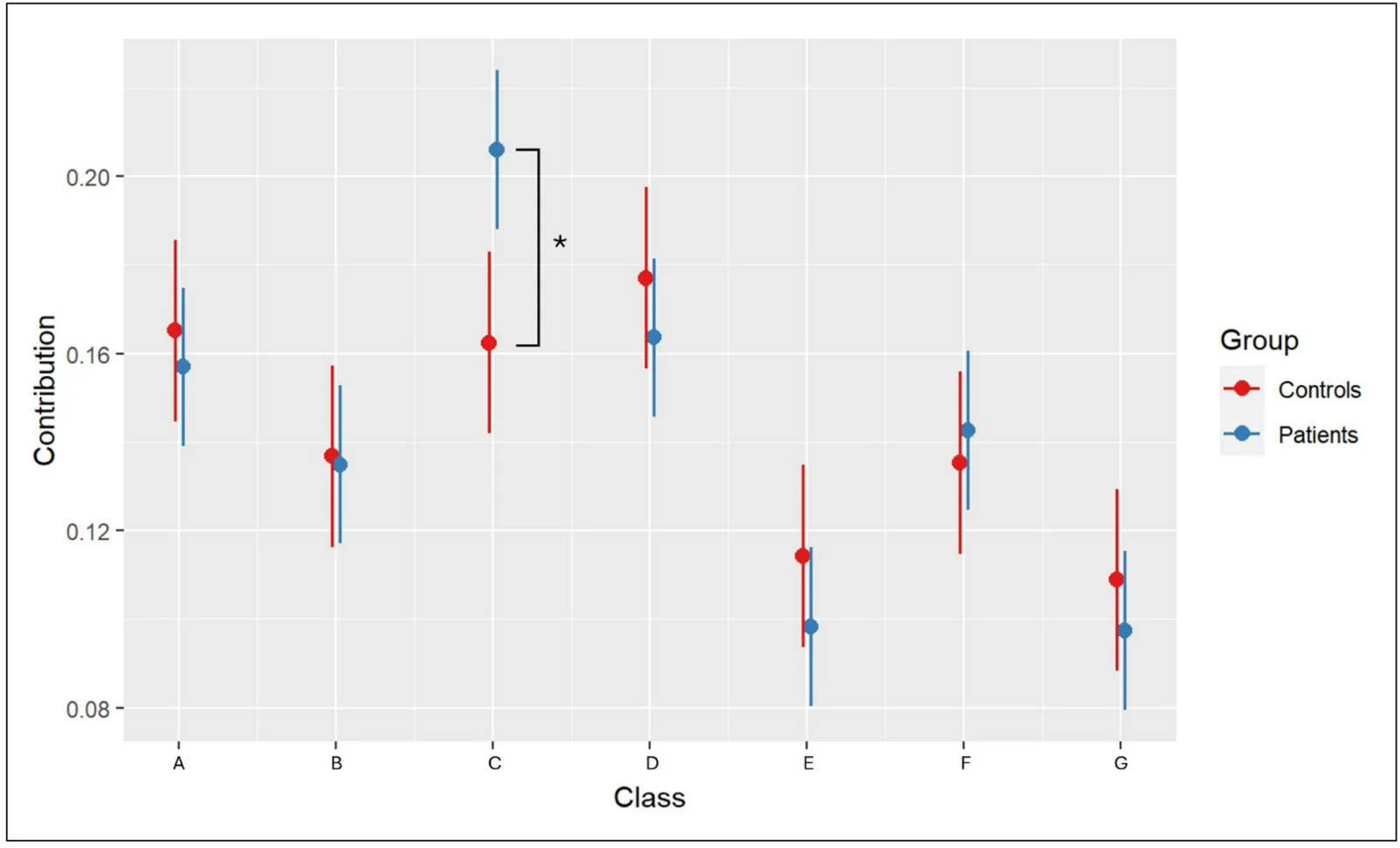
Group means and Standard Deviations of the MS parameter contribution. Patients with AUD showed significantly higher contribution values of MS C than HC

A significant main effect of *class* on contribution was found across all participants (*F*(6, 343) = 19.90, *p* < .001, η^2^_p_ = 0.26). Furthermore, a significant *class* x *group* interaction indicated that the contribution values of the different MS classes differed in dependence of the factor *group (F*(6, 343) = 2.23, *p* = .04, η^2^_p_ = 0.04). Further post-hoc ANOVAs showed that this interaction was based on a significant contrast in MS class C: Patients with AUD showed significantly higher contribution values (*t*_*Ratio*_(343) = -3.13, *p* = .002, d = -0.89) of the MS C than HC (see Fig. 2).

#### Scale-dependent Effects

We found significant three-way interactions of *class*, *group* and *scale* for “awareness of feelings” and “shame”. These results are described in detail below. No significant three-way interactions were found for any of the other ERSQ-ES or SEK-27 scales.

##### Awareness of Feelings

**Fig. 3:**
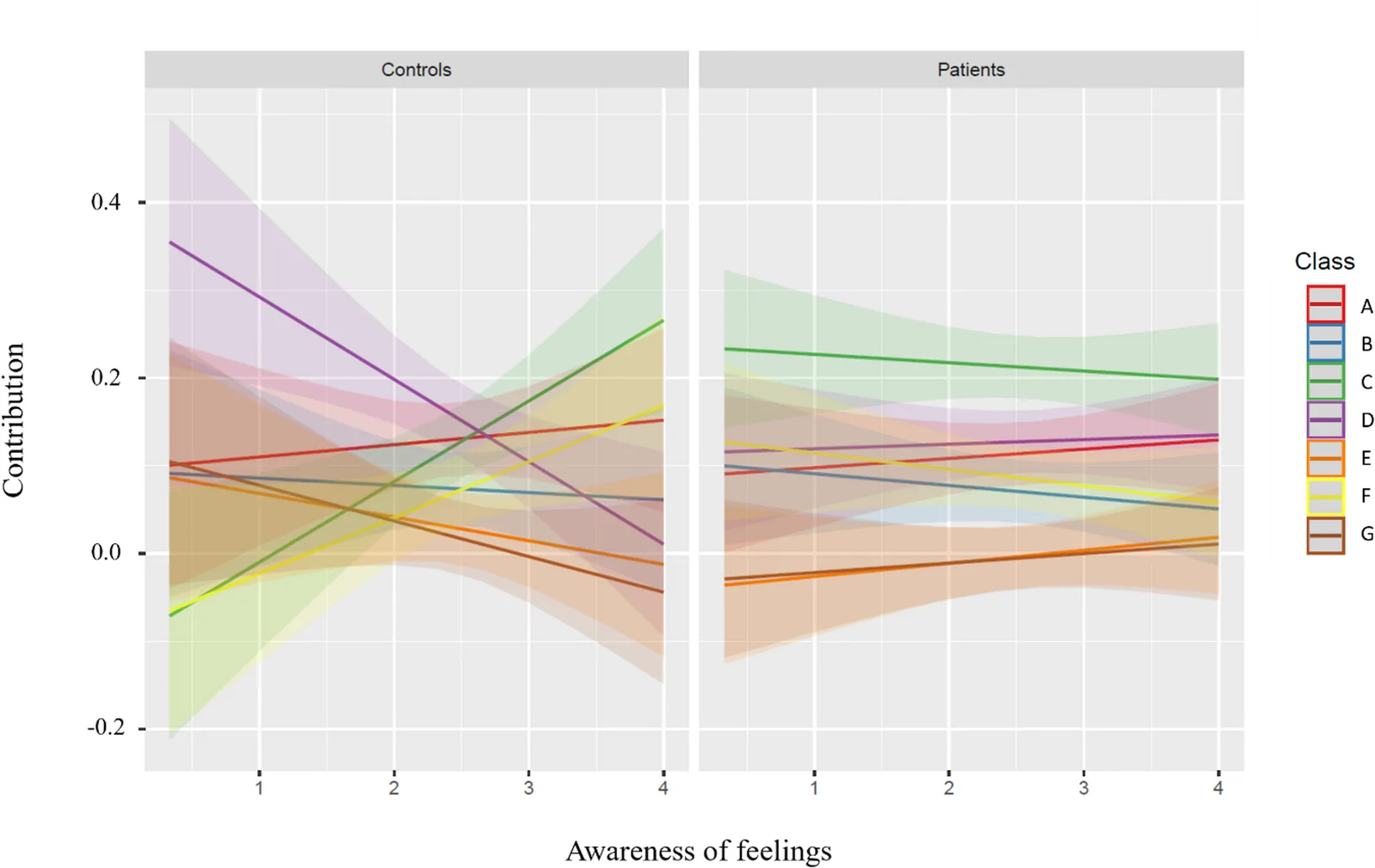
Predicted contribution values (y-axis) of the seven MSs (A-G) as a function of group and the score on the SEK-27 scale “awareness of feelings” (x-axis). Colored areas around the regression lines: Class-specific 95% confidence interval. In HC, higher “awareness of feelings” was associated with the higher contribution of MS C (green) and the lower contribution of MS D (purple)

*Three-way interaction (class x group x SEK-27 scale):* The results of the LMM regarding the MS parameter contribution showed a significant three-way interaction between *class*, *group* and *SEK-27 scale* (see Fig. 3): The contribution of the classes differed significantly depending on the group factor and the score on the scale “awareness of feelings” (*F*(6, 329) = 3.75, *p* = .001, η^2^_p_ = 0.06). The obtained *p*-value fell below the Bonferroni corrected alpha level (*α*_*Bonferroni*_ = 0.0056), so follow-up analyses were made using two-factorial, group-specific LMMs. These showed that the contribution of the MS classes differed significantly depending on the score of the scale “awareness of feelings” in HC (*F*(6, 140) = 4.30, *p* < .001, η^2^_p_ = 0.16) but not in patients with AUD (*F*(6, 189) = 0.48, *p* = .827, η^2^_p_ = 0.01). Additional Pearson correlations between the class-specific contribution values and the scores of “awareness of feelings” in the control group showed that the contribution of both MS C and D was significantly correlated with the score on the “awareness of feelings” scale. With *r*(20) = 0.48 (*p* = .025, r^2^ = 0.23), there was a positive correlation between the contribution of MS C and the score on the “awareness of feelings” scale, while an inverse correlation was found between the contribution of MS D and the score on the “awareness of feelings” scale (*r*(20) = − 0.47, *p* = .029, r^2^ = 0.22).

##### Shame

**Fig. 4:**
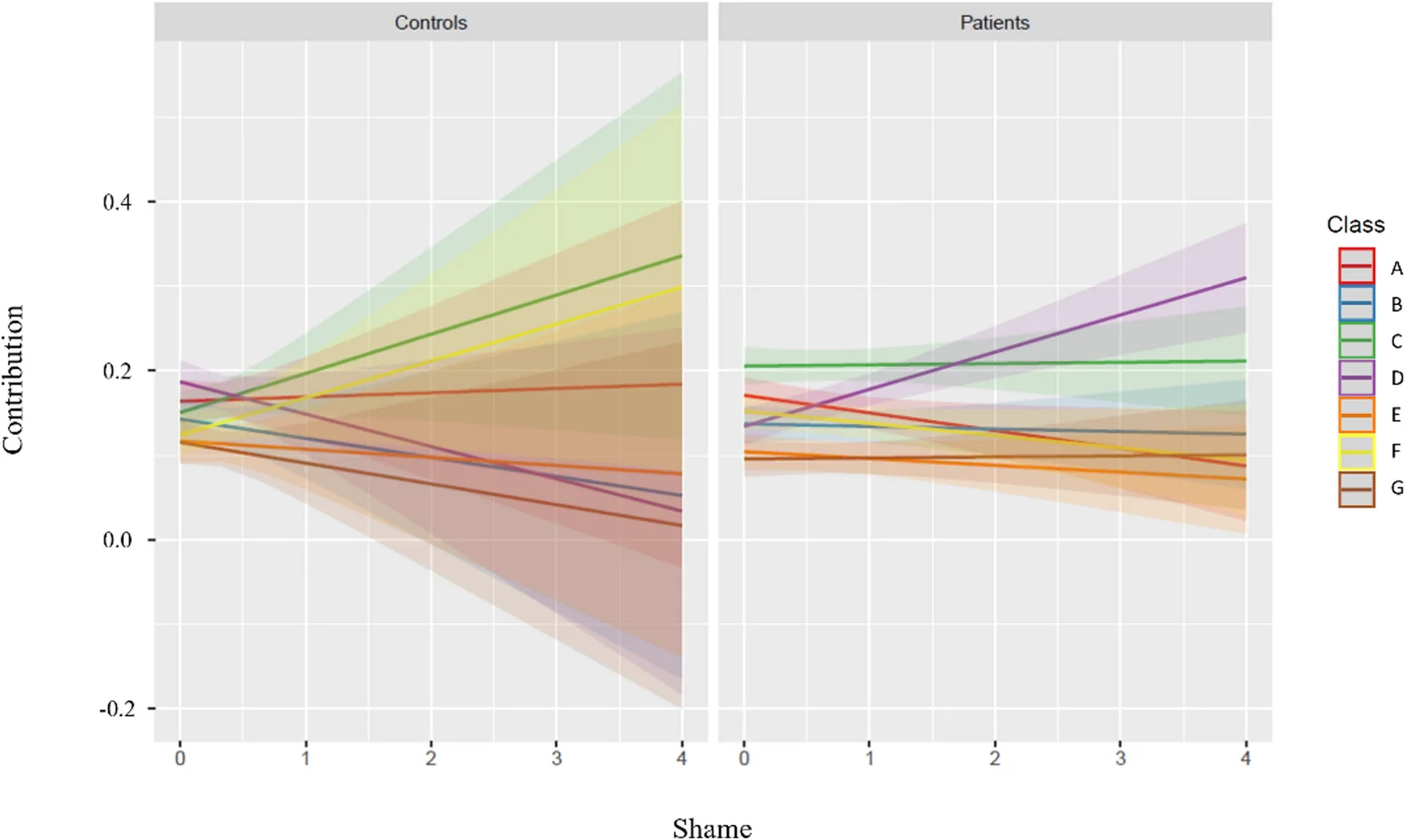
Predicted contribution values (y-axis) of the seven MSs (A-G) as a function of group and the score on the ERSQ-ES scale “shame” (x-axis). Colored areas around the regression lines: Class-specific 95% confidence interval. In patients with AUD, higher “shame” was associated with the lower contribution of MS A (red) and the higher contribution of MS D (purple)

*Three-way Interaction (class x Group x ERSQ-ES scale):* The results of the LMM regarding the MS parameter contribution showed a three-way interaction between *class*, *group* and *ERSQ-ES scale* (see Fig. 4), which did however not survive Bonferroni correction (*F*(6, 322) = 2.42, *p* = .026, η^2^_p_ = 0.04) and was therefore not considered significant and only reported for completeness. For exploration, the interaction was still tested using two-factorial, group-specific LMMs. With *F*(6, 182) = 5.05 (*p* < .001, η^2^_p_ = 0.14), it was found that the contribution of the MS classes differed significantly depending on the score of the scale “shame” in patients with AUD, while there was no such significant two-way interaction in HC (*F*(6, 140) = 1.27, *p* = .275, η^2^_p_ = 0.05). Following Pearson correlations between the class-specific contribution values and the group-specific (patients with AUD) scores of “shame”, showed a significant negative correlation between the contribution of MS A and the scale “shame” in patients with AUD (*r*(26) = − 0.50, *p* = .006, r^2^ = 0.25). Furthermore, a significant positive correlation was found between the contribution of MS D and “shame” in patients with AUD (*r*(26) = 0.65, *p* < .001, r^2^ = 0.42).

## Discussion

The present study investigated neurophysiological correlates of emotional processing in patients with AUD and HC using resting-state EEG data. The construct “emotional processing”, which was assessed with the questionnaires ERSQ-ES and SEK-27, includes feelings, moods, and emotional competencies. Regarding the seven MSs under investigation, the results indicate that the degree of the awareness to one‘s own feelings was associated with group-specific changes in temporal characteristics of the MS classes C and D. Primary analyses showed that the contribution of MS C was positively and the contribution of MS D negatively associated with the awareness to one’s own feelings in HC, while this effect was not observed in patients with AUD.

The results indicate that MS C is a pivotal neurophysiological marker for emotional processing, especially for awareness of emotions in HC. According to Tarailis et al. (2024) and Britz et al. (2010), MS C is associated with self-reflection, self-referential internal mentation and self-awareness. Since awareness to one’s own feelings requires high level of self-reflection, this assumption is supported by our results. The results also support the observed negative correlation between affective lability and the prevalence of MS C (Deiber et al. 2024). This might be explained by the fact that increased awareness to one’s own feelings leads to a more accurate assessment of internal emotional experiences, thereby enabling attempts to regulate emotional states (e.g. reappraisal, acceptance) and possibly resulting in emotional stability (Boden and Thompson 2015). With a topographical overlap of 94.0% between MS C in the present study and the study by Deiber et al. (2024), this finding seems especially relevant for the present study. Moreover, the present results can be reconciled with two studies by Zanesco et al. (2020, 2021) showing that increased neuroticism scores were associated with increased duration of MS C (Zanesco et al. 2020) and that mind wandering (vs. focusing on task) was associated with increased contribution of MS class C (Zanesco et al. 2021). With a stronger perception of unpleasant feelings being intrinsically included in the concept of awareness of feelings, individuals with higher awareness (and thus increased contribution of MS C) might display a stronger tendency to neuroticism and the focus on the inner world of an individual might favor mind wandering.

Contrary to this correlation between awareness of feelings and MS C contribution in HC, patients with AUD showed no such relationship, but – in the group comparison - a significantly higher contribution of MS C than HC. It thus seems that the linkage between awareness of feelings and MS C is not present in this clinical population, which is rather characterized by an overall elevated MS C contribution. One might argue that the imbalanced gender ratio might have impacted these results. However, Tomescu et al. (2018) reported higher duration of MS C in females than males and in our findings the AUD group (with fewer females) showed higher contribution of MS C than the HC group (with more females). Therefore, it is highly unlikely that our findings were driven by sex differences in resting EEG data.

MS D also seems to be an important marker for emotional processing. Our results show that higher contribution of MS D was associated with lower emotional awareness in HC (and potentially with higher feelings of shame in patients with AUD, although these are less robust (Bonferroni-uncorrected) results). These results support the findings that MS D is linked to deficient emotional processing. As Tarailis et al. (2021; topographical overlap: 92.5%) found, the self-domain of the ARSQ, with statements such as “I thought about my feelings”, was negatively correlated with the duration of MS D, which aligns with our findings. In our study, increased emotional awareness, likely similar to a higher ARSQ self-domain score, was associated with lower contribution of MS D in HC. Additionally, higher temporal dominance of MS D appears to be linked to intensified negative emotions. As found by Shen et al. (2020; topographical overlap: 90.0%) and Hu et al. (2023; topographical overlap: 95.1%), low emotional valence during video watching was associated with increased temporal dominance of MS D, which is consistent with our Bonferroni-uncorrected finding that higher feelings of shame were linked to higher contribution of MS D in patients with AUD. It is, however, less evident how these results align with a study by Zanesco et al. (2020), who found a positive correlation between MS D occurrence and the expression of the three personality dimensions of extraversion, agreeableness and conscientiousness. One may assume that high levels of extraversion, which focuses outwards, might be related to lower attention to and awareness of feelings (inward focus).

Further analyses regarding the ERSQ-ES scale “shame”, whose results should be interpreted with caution as the effects did not withstand the Bonferroni correction, indicated that the contribution of MS A was negatively correlated with the experienced shame in patients with AUD, but not in HC. This can be interpreted in light of the results of Schiller et al. (2020), who reported an association of increased prosociality with increased contribution of MS A. In the controversy about whether shame hinders or fosters prosociality (Li and Wang 2022; de Hooge et al. 2010; Feinberg et al. 2012), these results thus might indicate that the experience of shame might hinder prosocial behavior (Tangney et al. 1992, 1996).

There are some limitations to this study: Firstly, due to the lack of existing literature on neurophysiological activity and MS research in relation to emotion regulation abilities in patients with AUD, exploratory, not hypothesis-driven analyses were used. Secondly, it is important to note that the findings are based on correlations and do not imply causality. Thirdly, patients with AUD and HC differed regarding living situation and employment, thus introducing potential confounds. Fourthly, the inclusion of subjects who regularly consume nicotine could have led to a distortion of the MS results, since regular smoking led to changes in the temporal characteristics of the MSs in young men (Li et al. 2022). Finally, due to the exploratory nature of this study, no a-priori power analysis was conducted, which should be considered when interpreting the results.

In summary, this study investigates the temporal MS parameter contribution of seven MSs (A-G) in dependence on the group factor (patients with AUD vs. HC) and the emotional processing. The results show that the level of awareness of feelings and experienced shame (whose results should be treated with caution) are associated with group-specific temporal changes in MSs. In conclusion, these findings indicate that the neurophysiological basis of emotional processing (especially awareness of feelings) is impaired in patients with AUD when compared to HC. MS C, D (emotional awareness) and A (shame) emerge as critical components in this context. As both “shame” (Milan and Varescon 2024; Crozier et al. 2023) and “awareness of feelings” (Berking et al. 2011) are of high importance in the treatment of AUD, the neurophysiological deviations related to emotional processing may pose additional challenges for therapeutic approaches (e.g., emotion-focused interventions) in this population. As such, the present exploratory study contributes to MS research on emotional processing in HC and patients with AUD and serves to inform future, hypothesis-driven studies to generate new knowledge about the neurophysiological signature of emotional processing and its deviations in AUD.

## Supplementary Information

Below is the link to the electronic supplementary material.

Supplementary Material 1

## Data Availability

No datasets were generated or analysed during the current study.
