# Supplementary material for: EEG Microstate Correlates of Emotional Processing in Patients with Alcohol use Disorder and Healthy Controls: An Exploratory Resting-EEG Study
Source: Brain Topogr. 2026 Mar 27;39(3):37. doi: 10.1007/s10548-026-01180-z (PMC13658403; doi:10.1007/s10548-026-01180-z)
Supplement: Supplementary file 1 [file 10548_2026_1180_MOESM1_ESM.pdf]

Supplementary Information

EEG Microstate correlates of emotional processing in patients with alcohol use disorder and healthy controls: An exploratory resting-EEG study.

Oliver B. Affentranger*, Thomas Koenig, Franz Moggi, Leila M. Soravia, Ingo Butzke, Hallie M. Batschelet, Raphaela M. Tschuemperlin, Maria Stein.

Brain Topography | Springer
2025

This Supplementary Material has been provided by the authors to give readers additional details regarding the results of the study.

* Corresponding author at: Clinic Suedhang, Center for Treatment of Addictive Disorders, Südhang 1, 3038 Kirchlindach, Switzerland.
E-mail address: oliver.affentranger@gmail.com (O. B. Affentranger)

1 Results: additional 3

1.1 Additional results regarding the Microstate parameter "Duration": Linear (mixed) models and Pearson correlations 3

1.2 Additional results regarding the Microstate parameter "Occurrence": Linear (mixed) models and Pearson correlations 7

1.3 Additional results regarding the Microstate parameter "MeanGFP": Linear (mixed) models and Pearson correlations 11

1 Results: additional

1.1 Additional results regarding the Microstate parameter "Duration": Linear (mixed) models and Pearson correlations

We found a significant three-way interaction of *class*, *group* and *scale* for "awareness of feelings". No significant three-way interactions were found for any of the other ERSQ-ES or SEK-27 scales in the analyses of the microstate parameter duration. These results and the follow-up analyses are shortly summarized below.

	**p-values**		
ERSQ-ES/SEK-27 scale (regressor)	Linear mixed models	Linear models (follow-up)	Pearson correlations
**<u>Emotions and moods</u>**			
**Stress**	Three-way:<br>Class: < 0.001***<br><br>Two-way:<br>Class: < 0.001***	-	-
**Anxiety**	Three-way:<br>Class: < 0.001***<br><br>Two-way:<br>Class: < 0.001***	-	-
**Anger**	Three-way:<br>Class: < 0.001***<br>Class : Regressor: 0.022*<br><br>Two-way:<br>Class: < 0.001***<br>Class : Regressor: 0.013*	-	-
**Sadness**	Three-way:<br>Class: < 0.001***	-	-

	Two-way:<br>Class: < 0.001***		
**Depressiveness**	Three-way:<br>Class: < 0.001***<br><br>Two-way:<br>Class: < 0.001***	-	-
**Shame**	Three-way:<br>Class: < 0.001***<br>Class : Group: 0.008**<br><br>Two-way:<br>Class: < 0.001***<br>Class : Regressor: 0.002**	-	-
**Coping emotions**	Three-way:<br>Class: 0.043*<br>Class : Group: 0.041*<br>Class : Regressor: 0.028*<br><br>Two-way:<br>Class: 0.091	-	-
**Positive affect PANAS**	Three-way:<br>Class: 0.026*<br>Class : Group: 0.025*<br>Class : Regressor: 0.036*<br>Class : Group : Regressor: 0.069<br><br>Two-way:<br>Class: 0.057	AUD:<br>Class: 0.054<br><br>HC:<br>Class: 0.029*<br>Class : Regressor: 0.031*	-
**Negative affect PANAS**	Three-way:<br>Class: < 0.001***<br><br>Two-way:<br>Class: < 0.001***	-	-

<u>**Emotion processing and regulation**</u>			
**Awareness of feelings**	Three-way:<br>Class: 0.009**<br>Class : Group: 0.002**<br>Class : Regressor: 0.033*<br><u>**Class : Group : Regressor: 0.009**</u><br><br>Two-way:<br>Class: 0.026*	AUD:<br>Class: 0.018*<br><br>HC:<br>Class: 0.003**<br>**Class : Regressor: 0.004**	HC:<br><br>MS D:<br>r = -0.43<br>**p = 0.045***<br><br>MS G:<br>r = -0.36<br>p = 0.099
**Body sensation of feelings**	Three-way:<br>Class: 0.098<br>Regressor: 0.091<br><br>Two-way:<br>Class: 0.061<br>Regressor: 0.01	-	-
**Clarity of feelings**	Three-way:<br>Class: 0.020*<br><br>Two-way:<br>Class: 0.007**	-	-
**Understanding of feelings**	Three-way:<br>Group: 0.090<br>Regressor: 0.090<br><br>Two-way:<br>Class: 0.063	-	-
**Acceptance of feelings**	-	-	-
**Resilience**	Three-way:<br>Class : Group: 0.035*<br>Class : Regressor: 0.040*	AUD:<br>Class: 0.011*	-

	Class : Group : Regressor: 0.089<br><br>Two-way:<br>Class: 0.048*	HC:<br>Class : Regressor: 0.037*	
**Readiness to confront**	Three-way:<br>Class: 0.002**<br>Class : Regressor: 0.004**<br><br>Two-way:<br>Class: 0.008**<br>Class : Regressor: 0.021*	-	-
**Self-support**	Three-way:<br>Class: 0.040*<br><br>Two-way:<br>Class: 0.044*	-	-
**Regulatory competence**	Three-way:<br>Class : Group: 0.027*<br>Class : Regressor: 0.052<br>Class : Group : Regressor: 0.089	HC:<br>Class: 0.061<br>Class : Regressor: 0.036*	-
**Emotion regulation (total scale)**	Three-way:<br>Class: 0.060<br>Class : Regressor: 0.045*	-	-

Notes. Sample: $N = 51$. Reported results: Only trends ($p < .10$) and significant results ($p < .05$; significance level: $\alpha = .05$) are shown. Results that should not be considered due to prior computations are highlighted in gray. Abbreviations: AUD = Alcohol use disorder; Class = Microstate classes; Regressor = Score on a specific ERSQ-ES/SEK-27 scale; Three-way = Linear mixed model with three factors (class, group, regressor); Two-way = Linear mixed model with two factors (class, regressor); r = Pearson product–moment correlation.
Note on probability. * $p < .05$. ** $p < .01$. *** $p < .001$.

1.2 Additional results regarding the Microstate parameter “Occurrence”: Linear (mixed) models and Pearson correlations

We found significant three-way interactions of *class*, *group* and *scale* for “shame” and “awareness of feelings”. No significant three-way interactions were found for any of the other ERSQ-ES or SEK-27 scales in the analyses of the microstate parameter occurrence. These results and the follow-up analyses are shortly summarized below.

	**p-values**		
ERSQ-ES/SEK-27 scale (regressor)	Linear mixed models	Linear models (follow-up)	Pearson correlations
**Emotions and moods**			
**Stress**	Three-way:<br>Class: < 0.001***<br><br>Two-way:<br>Class: < 0.001***	-	-
**Anxiety**	Three-way :<br>Class: < 0.001***<br>Class : Group: 0.046*<br><br>Two-way:<br>Class: < 0.001***	-	-
**Anger**	Three-way:<br>Class: 0.001**<br><br>Two-way:<br>Class: < 0.001***	-	-
**Sadness**	Three-way:<br>Class: < 0.001***<br>Class : Group: 0.061<br><br>Two-way:<br>Class: < 0.001***	-	-

**Depressiveness**	Three-way:<br>Class: < 0.001***<br><br>Two-way:<br>Class: < 0.001***	-	-
**Shame**	Three-way:<br>Class: < 0.001***<br>Class : Group: 0.002**<br>**Class : Group : Regressor: 0.033***<br><br>Two-way:<br>Class: < 0.001***<br>Class : Regressor: 0.061	AUD:<br>Class: < 0.001***<br>**Class : Regressor: 0.01***<br><br>HC:<br>Class: < 0.001***<br><br>MS A:<br>Regressor: 0.007**<br><br>MS C:<br>Group: 0.027*<br><br>MS D:<br>Group : Regressor: 0.032*	AUD:<br><br>MS A:<br>r = -0.49<br>**p = 0.008****<br><br>MS D:<br>r = 0.39<br>**p = 0.041***
**Coping emotions**	Three-way:<br>Class : Group: 0.058	-	-
**Positive affect PANAS**	Two-way:<br>Class: 0.057	-	-
**Negative affect PANAS**	Three-way:<br>Class: < 0.001***<br>Class : Group: 0.009**<br>Class : Group : Regressor: 0.070<br><br>Two-way:<br>Class: < 0.001***	AUD:<br>Class: < 0.001***<br><br>HC:<br>Class: 0.034*<br><br>MS C:<br>Group: 0.022*<br><br>MS D:<br>Group : Regressor: 0.026*	-

		MS G:<br>Group : Regressor: 0.058	
**Emotion processing and regulation**			
**Awareness of feelings**	Three-way:<br>Class : Group: < 0.001***<br>**Class : Group : Regressor: 0.007****	AUD:<br>Class: 0.006**<br><br>HC:<br>Class: 0.009**<br>**Class : Regressor: 0.007****<br><br>MS C:<br>Group: 0.015*<br>Group : Regressor: 0.019*	HC:<br><br>MS C:<br>r = 0.58<br>**p = 0.004****<br><br>MS F:<br>r = 0.37<br>p = 0.095
**Body sensation of feelings**	Three-way:<br>Class : Group: 0.058	-	-
**Clarity of feelings**	Two-way:<br>Class: 0.047*	-	-
**Understanding of feelings**	-	-	-
**Acceptance of feelings**	-	-	-
**Resilience**	-	-	-
**Readiness to confront**	-	-	-

**Self-support**	-	-	-
**Regulatory competence**	-	-	-
**Emotion regulation (total scale)**	-	-	-

Notes. Sample: $N = 51$. Reported results: Only trends ($p < .10$) and significant results ($p < .05$; significance level: $\alpha = .05$) are shown. Results that should not be considered due to prior computations are highlighted in gray. Abbreviations: AUD = Alcohol use disorder; Class = Microstate classes; Regressor = Score on a specific ERSQ-ES/SEK-27 scale; Three-way = Linear mixed model with three factors (class, group, regressor); Two-way = Linear mixed model with two factors (class, regressor); *r* = Pearson product–moment correlation.
Note on probability. * $p < .05$. ** $p < .01$. *** $p < .001$.

1.3 Additional results regarding the Microstate parameter “MeanGFP”: Linear (mixed) models and Pearson correlations

We found a significant three-way interaction of *class*, *group* and *scale* for “awareness of feelings”. No significant three-way interactions were found for any of the other ERSQ-ES or SEK-27 scales in the analyses of the microstate parameter MeanGFP. These results and the follow-up analyses are shortly summarized below.

	**p-values**		
ERSQ-ES/SEK-27 scale (regressor)	Linear mixed models	Linear models (follow-up)	Pearson correlations
**Emotions and moods**			
**Stress**	Three-way:<br>Class: < 0.001***<br><br>Two-way:<br>Class: < 0.001***	-	-
**Anxiety**	Three-way:<br>Class: < 0.001***<br><br>Two-way:<br>Class: < 0.001***	-	-
**Anger**	Three-way:<br>Class: < 0.001***<br><br>Two-way:<br>Class: < 0.001***	-	-
**Sadness**	Three-way:<br>Class: < 0.001***<br><br>Two-way:<br>Class: < 0.001***	-	-
**Depressiveness**	Three-way:<br>Class: < 0.001***	-	-

	Two-way:<br>Class: < 0.001***		
**Shame**	Three-way:<br>Class: < 0.001***<br>Class : Group: 0.028*<br><br>Two-way:<br>Class: < 0.001***	-	-
**Coping emotions**	Two-way:<br>Class: 0.008**	-	-
**Positive affect PANAS**	Two-way:<br>Class: < 0.001***	-	-
**Negative affect PANAS**	Three-way:<br>Class: < 0.001***<br><br>Two-way:<br>Class: < 0.001***	-	-
**<u>Emotion processing and regulation</u>**			
**Awareness of feelings**	Three-way:<br>Class: < 0.001***<br>Class : Group: 0.012*<br>**<u>Class : Group : Regressor: 0.038*</u>**<br><br>Two-way:<br>Class: < 0.001***	AUD:<br>Class: < 0.001***	-
**Body sensation of feelings**	Three-way:<br>Class: < 0.001***<br><br>Two-way:<br>Class: < 0.001***	-	-

**Clarity of feelings**	Three-way:<br>Class: 0.003**<br><br>Two-way:<br>Class: < 0.001***	-	-
**Understanding of feelings**	Three-way:<br>Class: 0.032*<br><br>Two-way:<br>Class: < 0.001***	-	-
**Acceptance of feelings**	Three-way:<br>Class: 0.036*<br><br>Two-way:<br>Class: < 0.001***	-	-
**Resilience**	Two-way:<br>Class: < 0.001***	-	-
**Readiness to confront**	Three-way:<br>Class: 0.032*<br><br>Two-way:<br>Class: 0.007**	-	-
**Self-support**	Three-way:<br>Class: < 0.001***<br><br>Two-way:<br>Class: < 0.001***	-	-
**Regulatory competence**	Three-way:<br>Class: 0.056<br><br>Two-way:<br>Class: < 0.001***	-	-

**Emotion regulation (total score)**	Two-way:<br>Class: 0.003**	-	-

Notes. Sample: $N = 51$. Reported results: Only trends ($p < .10$) and significant results ($p < .05$; significance level: $\alpha = .05$) are shown. Results that should not be considered due to prior computations are highlighted in gray. Abbreviations: AUD = Alcohol use disorder; Class = Microstate classes; Regressor = Score on a specific ERSQ-ES/SEK-27 scale; Three-way = Linear mixed model with three factors (class, group, regressor); Two-way = Linear mixed model with two factors (class, regressor); *r* = Pearson product–moment correlation.
Note on probability. * $p < .05$. ** p < .01. *** $p < .001$.
